# Influence of the Ionic Liquid on the Activity of a Supported Ionic Liquid Phase Fe^II^ Pincer Catalyst for the Hydrogenation of Aldehydes

**DOI:** 10.1002/ejic.201900636

**Published:** 2019-07-29

**Authors:** Zita Csendes, Julian Brünig, Nevzat Yigit, Günther Rupprechter, Katharina Bica‐Schröder, Helmuth Hoffmann, Karl Kirchner

**Affiliations:** ^1^ Institute of Applied Synthetic Chemistry Vienna University of Technology Getreidemarkt 9 A‐1060 Vienna Austria; ^2^ Institute of Materials Chemistry Vienna University of Technology Getreidemarkt 9 A‐1060 Vienna Austria

**Keywords:** Hydrogenation, Ionic liquids, Iron, Supported catalysts, Sustainable chemistry

## Abstract

The catalytic hydrogenation of different aldehydes to the corresponding alcohols was investigated using an Fe^II^ hydride pincer complex as catalyst in the supported ionic liquid phase (SILP) reaction mode. Two different ionic liquids of the type [X_4441_][NTf_2_] with X=N or P were applied with mesoporous silica gel as support, which was coated first with a chemisorbed monolayer of the corresponding modified IL to remove acidic surface OH‐groups and to prevent IL leaching. Quantitative conversion with turn‐over frequencies in the order of 1000 h^–^^1^ were obtained for various aromatic and heteroaromatic aldehydes and highly selective aldehyde reduction was observed also for substrates containing reducible C=C bonds. Aldehydes with longer aliphatic chains or cycloalkyl substituents, however, showed no conversion here, in contrast to a previous study with an imidazolium‐based ionic liquid. These differences were ascribed primarily to differences in substrate/ionic liquid interactions. Whereas [N_4441_][NTf_2_] and [P_4441_][NTf_2_] gave essentially identical results for different substrates in single‐batch reactions, prolonged use of the catalyst in repeated reaction cycles lead to a quick drop‐off in catalyst activity in [P_4441_][NTf_2_], but a continuous, quantitative conversion in [N_4441_][NTf_2_].

## Introduction

Over the past 15 years, supported ionic liquid phase (SILP) catalysis has emerged as a powerful hybrid technology of classical homogeneous and heterogeneous reaction techniques^1^ Therein, the catalyst is dissolved in an ionic liquid and is applied as a thin film coated onto a high surface area support material. This liquid‐impregnated support can be applied just like a heterogeneous catalyst either as a powder, a packed cartridge or a monolithic structure and contains the catalyst in an easily separable and reusable phase. The catalyst itself is dissolved in the ionic liquid and is believed to retain its chemical nature, its activity, and selectivity analogous to a classical, homogeneous environment. Previous studies have shown, however, that this assumption is rarely fulfilled and that the unique multiphase environment of a SILP catalyst entails several peculiarities which influence the catalyst's performance: First, ionic liquids are highly polar liquids, which tend to display complex structural patterns at or near interfaces such as domain formation, surface segregation, solvent cages etc.^2^ The ionic liquid layer of a SILP catalyst which is typically only a few nanometers thick can therefore hardly be considered as a bulk solution phase and an inert reaction medium like the solvents that are typically used in homogeneous catalytic reactions. Second, physical support properties like pore size, pore‐volume, and wetting properties determine how the ionic liquid film spreads across the support surface and how well it adheres to the support.^3^ Third, unfavorable cross solubilities of catalyst and ionic liquid in the reactant solution and of substrate and product in the ionic liquid layer can shift the reaction equilibrium and slow down or stop the reaction. Fourth, functional groups at the support surface might interact or react with the catalyst and influence its activity.^4^ The knowledge of how the catalyst and the reaction mechanism are influenced by these parameters is still very limited and requires more comparative studies of catalytic reactions under homogeneous and SILP reaction conditions.

We have recently started to expand our studies on base metal pincer catalysts in homogeneous solution^5^ to IL‐based multiphase reaction techniques such as liquid‐liquid biphasic^6^ and SILP catalysis.^7^ As a test reaction, the reduction of aldehydes to alcohols using [Fe(PNP^Me^‐*i*Pr)(CO)(H)(Br)] as catalyst was used. In the biphasic mode,^6^ the catalyst was dissolved in various ionic liquids A^+^ [NTf_2_]^–^ with the weakly coordinating [NTf_2_]^–^ anion together with different types of cations A^+^ (ammonium, phosphonium, imidazolium, pyridinium, picolinium as well as targeted Bronsted acidic species) and was brought in contact with a second, immiscible organic solvent phase which contained the substrate. In the SILP mode,^7^ the catalyst was dissolved in 1‐butyl‐2,3‐dimethyl‐imidazolium bis(trifluoromethylsulfonyl)imide ([bm_2_im][NTf_2_]) and was applied as a thin layer coated onto two different support materials: silica[7a] and carbon.[7b]

In general, the chemoselectivity of this catalyst for the reduction of aldehydes was preserved and C=C double bonds or C=O ketone groups were not reduced in both the biphasic and the SILP mode. However, the catalytic activity and, in particular, the reaction rates (TOF numbers) were lower than the homogeneous reactions and depended strongly on the type of substrate, the support pretreatment and the particular ionic liquid used.

Silica supports had to be coated first with a chemisorbed layer of ionic liquid in order to remove acidic surface hydroxyl groups which destroyed the catalyst. Carbon supports could be used without pretreatment but showed 4–10 times lower reaction rates due to their smaller pore sizes and the thereby reduced active area of the ionic liquid film. The influence of the ionic liquid on the catalytic activity was initially investigated in the biphasic reaction mode. Isolated yields were in the range from < 1 % to > 99 % and showed that the ionic liquid has a tremendous influence on the catalytic function. The best results were obtained with [P_4441_][NTf_2_] which yielded complete conversion at a turn over frequency of 1330 h^–^^1^ compared to 2000 h^–^^1^ for the homogeneous reaction mode under otherwise equal conditions. One drawback of the biphasic mode, however, is the relatively large amount of ionic liquid that is needed, which dissolves a substantial part of the produced alcohol. The higher solubility of the formed alcohol compared to the starting materials complicated work‐up, and quantitative recovery of the reaction product, therefore, requires subsequent extraction of the IL phase and prevents a continuous operation in this biphasic configuration.

In the SILP mode, in comparison, the total amount of ionic liquid is much smaller and product losses due to dissolution in the ionic liquid phase are usually negligible. For this work, we, therefore, chose two ionic liquids – [P_4441_][NTf_2_] and [N_4441_][NTf_2_] – which gave the best results in the biphasic mode and tested them for SILP catalysis. By comparison with the biphasic data and with previous SILP data using an imidazolium‐based ionic liquid, we were hoping to gain deeper insights into the role of the ionic liquid in this model reaction.

## Results and Discussion

### Surface Modification of Silica Gel

**Figure 1:**
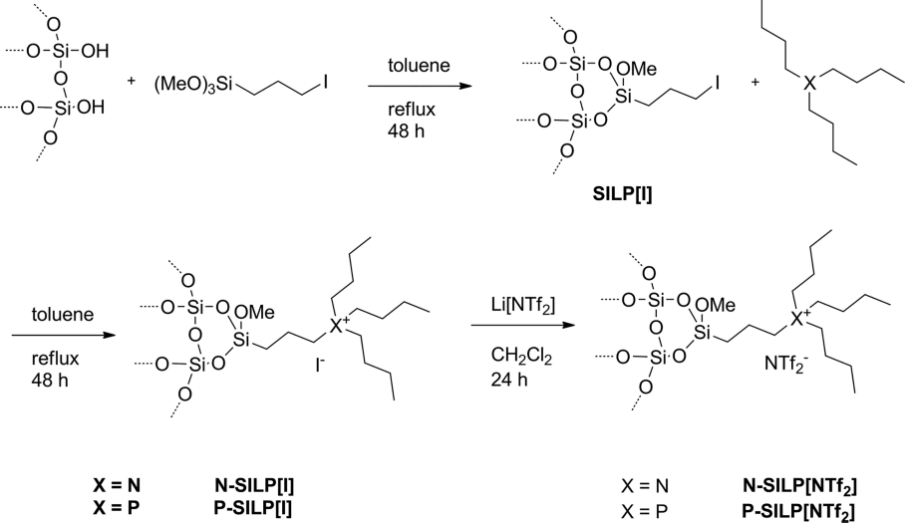
Step by step functionalization of silica gel.

The hydride ligand in the catalyst precursor **1**, as well as the hydride ligands of the in situ generated active catalyst [Fe(PNP^Me^‐*i*Pr)(CO)(H)_2_] (**2**), are strongly basic and thus sensitive to acidic functionalities such as the OH groups of the silica gel. To avoid catalyst decomposition by this group and to make the surface of silica gel “IL‐philic” to prevent IL leaching, the silica gel was covalently coated, in a three‐step procedure, with the corresponding IL (Figure 1). In the first step, 3‐iodopropyltrimethoxysilane was covalently grafted yielding SILP[I]. Then, SILP[I] was treated with tributylamine or tributylphosphine to yield N‐SILP[I] and P‐SILP[I], respectively. Finally, N‐SILP[I] and P‐SILP[I] were treated with Li[NTf_2_] to obtain N‐SILP[NTf_2_] and P‐SILP[NTf_2_].

From elemental analysis of the nitrogen content, the ionic liquid loadings of the ammonium‐ and phosphonium‐functionalized silica gels were calculated as 0.22 mmol/g (N‐SILP[NTf_2_]) and 0.25 mmol/g (P‐SILP[NTf_2_]). With the corresponding BET surface areas (Table 1) the mean surface coverage is about 4 × 10^13^ molecules/cm^2^ for N‐SILP[NTf_2_] and around 6 × 10^13^ molecules/cm^2^ for P‐SILP[NTf_2_]. These values are about one order of magnitude lower than the densest packing of 5 × 10^14^ molecules/cm^2^ found in long‐chain *n*‐alkylsiloxane monolayers on silica.^8^ This must be ascribed to the rather bulky ammonium and phosphonium cations in the present system.

**Table 1:** Structural parameters calculated from the N_2_ adsorption‐desorption isotherms

Sample	BET	Pore	Average	*^ α ^*
	surface	volume^b^	pore	
	area^a^	[cm^3^/g]	diameter^c^	[%]^d^
	[m^2^/g]			
N‐SILP[NTf_2_]	325	0.50	5.4	
N‐SILP10	138	0.26	4.8	17
N‐SILP20	66	0.15	5.2	38
N‐SILP30	4	0.02	14.0	65
N‐SILP40	too low to measure			≈ 100
P‐SILP[NTf_2_]	269	0.39	4.3	
P‐SILP10	112	0.21	4.7	21
P‐SILP20	55	0.12	5.0	48
P‐SILP30	2	0.01	16.9	83
P‐SILP40	too low to measure			≈ 100

a Calculated by the BET equation.

b BJH pore desorption volume.

c Desorption average pore diameter.

d Pore filling degree (IL volume/pore volume) × 100.

**Figure 2:**
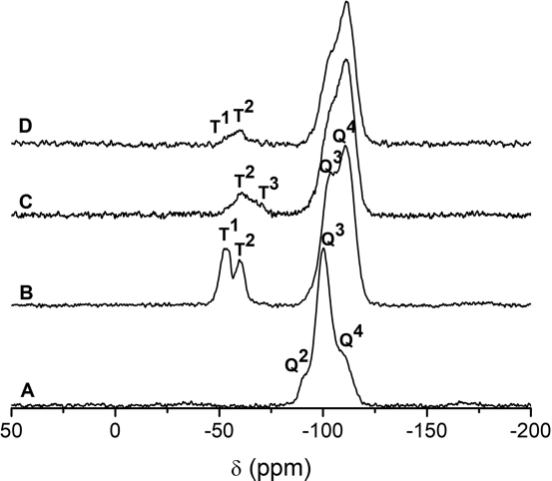
Solid‐state ^29^Si CP‐MAS NMR spectra of (A) pristine SG, (B) SILP[I], (C) N‐SILP[I] and (D) P‐SILP[I].

The surface modification sequence depicted in Figure 1 was studied by ^29^Si{^1^H}CP‐MAS NMR spectroscopy (Figure 2). Pristine SG (Figure 2A) displays three resonances at –110, –100 and –91 ppm which are characteristic of Si(OSi)_4_ (Q^4^), Si(OSi)_3_OH (Q^3^) and Si(OSi)_2_(OH)_2_ (Q^2^) species of the silica framework, respectively.^9^ Anchoring 3‐iodopropyltrimethoxysilane results in a reduction of the Q^3^ and Q^2^ bands (Figure 2B), and a concomitant relative increase of the Q^4^ peak. This indicates the reaction of singular and geminal silanol groups with the trimethoxysilane moiety. The spectrum of SILP[I] in Figure 2B also displays two signals at –60 and –53 ppm, which clearly confirm that the organic molecule is covalently grafted to the silica surface via either two RSi(OSi)_2_(OMe)(T^2^) or one RSi(OSi)(OMe)_2_ (T^1^) siloxane bonds, respectively.

No resonances appeared above –50 ppm, indicating the absence of physically adsorbed silane molecules. The solid‐state ^29^Si NMR spectrum of N‐SILP[I] (Figure 2C) revealed that reacting SILP[I] with tributylamine further promoted the direct condensation of the grafted siloxy functionalized moiety with silica since the T^1^ signal at –53 ppm disappeared while the T^3^ [RSi(OSi)_3_] signal at –68 ppm appeared in the spectrum. It means that the functionalized ionic liquid is grafted via two or three siloxane bonds to the surface of the support. The ^29^Si NMR spectrum of P‐SILP[I] (Figure 2D), on the other hand, shows only a decrease of the T^1^ peak compared to SILP[I], but no T^3^ signal of triple‐bonded RSi(OSi)_3_ moieties, indicating that tributylphosphine did not promote the above mentioned further condensation as efficiently as tributylamine. This observation can be explained by the higher basicity of N(Bu)_3_ (p*K*_a_ = 10.9)^10^ compared to P(Bu)_3_ (p*K*_a_ = 8.4).^11^ Similar effects were observed previously with trimethylamine and pyridine as base catalysts of surface silanol condensation.^12^

**Figure 3:**
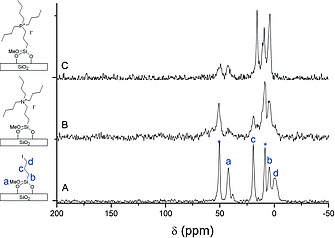
Solid‐state ^13^C CP‐MAS NMR spectrum of (A) SILP[I], (B) N‐SILP[I], and (C) P‐SILP[I].

^13^C CP‐MAS NMR spectra also confirm the successful modification of the surface of silica gel (Figure 3). The spectrum of SILP[I] displays peaks at –0.5 (d), 4.6 (b), 19.2 (c) and 42.1 (a) ppm that are assigned as (d) the carbon next to I, (b) the carbon next to Si, (c) the center carbon of the C3 chain, and (a) the carbon in the methoxy group. The peaks marked with asterisks are attributed to ethanol residue. The shift of the peak at –0.5 (d) ppm to the 4–24 ppm region in the spectra of N‐SILP[I] (Figure 3B) and P‐SILP[I] (Figure 3C) proves the displacement of iodine^13^ and thus the reaction of the SiO_2_‐supported propyl iodide with tributylamine and tributylphosphine with formation of a monolayer of ammonium‐ and phosphonium‐based ionic liquid on the surface of the support. The decrease of the peak intensity of the methoxy carbon in the spectra 3B and 3C also confirms that further condensation/hydrolysis took place catalyzed by the basic N(Bu)_3_ and P(Bu)_3_ reactants. The peaks of the propyl and butyl carbon atoms between 4–24 ppm in the spectra of N‐SILP[I] and P‐SILP[I] are broadened and not well‐resolved. The ion exchange of I^–^ to [NTf_2_]^–^ did not change the NMR spectra (not shown). ^31^P CP‐MAS NMR spectroscopy also confirmed the successful formation of the phosphonium‐based IL layer on the surface since the corresponding peak at 30 ppm can be found in the spectrum (not shown).

**Figure 4:**
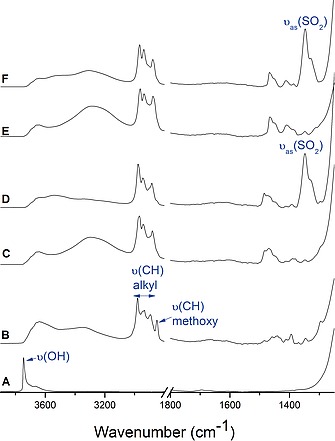
FT‐IR spectrum of (A) SG, (B) SILP[I], (C) N‐SILP[I], (D) N‐SILP[NTf_2_], (E) P‐SILP[I], and (F) P‐SILP[NTf_2_].

Additional evidence for the successful modification of the silica support is provided by the infrared spectra shown in Figure 4. Silica gel pretreated at 400 °C yields a spectrum with just one, highly characteristic absorption at 3748 cm^–1^ in Figure 4A due to the OH stretching vibration of isolated surface hydroxyl groups. This absorption disappears, as expected, upon adsorption of iodopropyl trimethoxysilane (Figure 4B), which indicates the removal of these acidic OH‐groups. Concurrently, the ν(CH) stretching absorptions of the propyl group between 3035 cm^–1^ and 2875 cm^–1^ appear in the spectrum of Figure 4B together with a characteristic single peak at 2850 cm^–1^ due to the ν(CH_3_) vibration of the OCH_3_ groups.^14^ The latter absorption indicates incomplete hydrolysis and condensation of the –Si(OCH_3_)_3_ anchor groups, in agreement with the previously discussed ^29^Si and ^13^C NMR spectra. The subsequent coupling of N(Bu)_3_ and P(Bu)_3_, respectively, to the surface (spectra 4C and 4E) yields changes in the ν(CH) stretching absorptions around 3000 cm^–1^ and the δ(CH) deformation modes (1400 cm^–1^–1500 cm^–1^) and, most noticeably, the disappearance of the methoxy ν(CH_3_) absorption at 2850 cm^–1^, indicating a base‐catalyzed progression of methoxy group hydrolysis and condensation. This has also been attested before in the ^29^Si and ^13^C NMR spectra. Finally, the ion exchange step [I]^–^ → [NTf_2_]^–^ is evidenced by the intense absorption of ν_as_(SO_2_) at 1348 cm^–1^ and ν_as_(CF_3_) at 1196 cm^–1^.^15^

### Impregnation of Modified Silica Gel with Ionic Liquid/Catalyst

**Figure 5:**
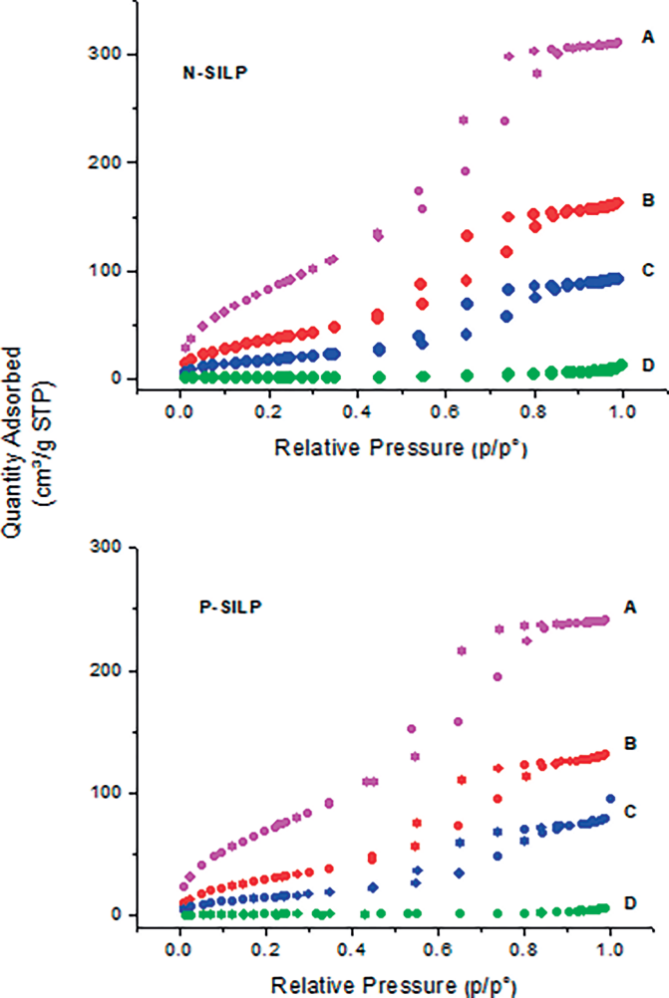
N_2_ adsorption–desorption isotherms of N‐SILP and P‐SILP catalysts with different ionic liquid loadings *ε*. A) SILP[NTf_2_], *ε* = 0, B) SILP10, *ε* = 10 %, C) SILP20, *ε* = 20 %, D) SILP30, *ε* = 30 %.

In order to find the optimum pore filling, N‐SILP[NTf_2_] and P‐SILP[NTf_2_] were impregnated with 10, 20, 30, and 40 wt.‐% of **1** dissolved in ([N_4441_][NTf_2_]) and ([P_4441_][NTf_2_]) producing N‐SILP10, N‐SILP20, N‐SILP30, N‐SILP40 and P‐SILP10, P‐SILP20, P‐SILP30, P‐SILP40, respectively. These materials were characterized by N_2_ adsorption‐desorption (Figure 5). The obtained isotherms are type IV isotherms according to the IUPAC classification with capillary condensation taking place between *p/p*_0_ =0.5 and *p/p*_0_ =0.8, characteristic of mesoporous solids with pore diameters between 2 nm and 50 nm. N‐SILP40 and P‐SILP40 could not be measured due to the low surface area.

Structural parameters derived from N_2_ adsorption‐desorption isotherms showed that the pore volume continuously decreased upon loading with IL/catalyst solution due to the filling of the pores (Table 1). The pore filling degree (*α*) increases with the IL loading up to 100 % for SILP40s.

### Catalytic Aldehyde Hydrogenation

The hydrogenation of 4‐fluorobenzaldehyde to 4‐fluorobenzyl alcohol was used as a test reaction to investigate the performance of SILP catalysis with two different ionic liquids [N_4441_][NTf_2_] and [P_4441_][NTf_2_]. 50 bar H_2_ pressure, 5 mol‐% of DBU (1,8‐diaza‐bicyclo[5.4.0]undec‐7‐ene) as a base in *n*‐heptane at 25 °C were applied. The results are shown in Table 2. The reaction times were chosen to achieve quantitative conversion for each entry. No conversion was obtained without a catalyst. The N‐SILP and the P‐SILP systems showed essentially equal performance. An approximately linear decrease of reaction time and a corresponding increase of TOFs with IL loading was observed (entries 1–4 and 6–9). The SILP 40 systems, however, which gave the highest TOF values (entries 4 and 9), showed substantial leaching of both the catalyst and the ionic liquid into the *n*‐heptane solution, as evidenced from ICP‐OES and ^19^F{^1^H} NMR measurements. In that case, partial homogeneous hydrogenation in the *n*‐heptane solution is likely the reason for the high reaction rates.

**Table 2:** SILP hydrogenation of 4‐fluorobenzaldehyde in different ionic liquids: [N_4441_][NTf_2_] (N‐SILP), [P_4441_][NTf_2_] (P‐SILP) and [bm_2_im][NTf_2_] (Im‐SILP)

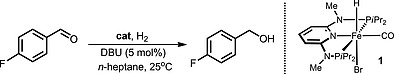								
Entry	Conditions^a^	S/C	P	Time	Conversion	TON	TOF	IL leaching
			[bar]	[min]	[%]		(h^–1^)	
1	N‐SILP10	200	50	20	> 99	200	600	no
2	N‐SILP20	200	50	15	> 99	200	800	no
3	N‐SILP30	200	50	15	> 99	200	800	no
4	N‐SILP40	200	50	5	> 99	200	2400	yes
5	N‐SILP20	1000	50	45	> 99	1000	1333	no
6	P‐SILP10	200	50	20	> 99	200	600	no
7	P‐SILP20	200	50	15	> 99	200	800	no
8	P‐SILP30	200	50	10	> 99	200	1200	no
9	P‐SILP40	200	50	10	> 99	200	1200	yes
10	P‐SILP20	1000	50	45	> 99	1000	1333	no
11	Im‐SILP20	1000	50	15	> 99	1000	4000	no

a Conditions: entry 1,6: 2 mmol substrate, 1000 mg of SILP10 (5 mg of **1**, 95 mg of IL, 900 mg of SILP[NTf_2_]); entry 2,7: 2 mmol substrate, 500 mg of SILP20 (5 mg of **1**, 95 mg of IL, 400 mg of SILP[NTf_2_]); entry 3,8: 2 mmol substrate, 333 mg of SILP30 (5 mg of **1**, 95 mg of IL, 233 mg of SILP[NTf_2_]); entry 4,9: 2 mmol substrate, 250 mg of SILP40 (5 mg of **1**, 95 mg of IL, 150 mg of SILP[NTf_2_]); entry 5,10: 10 mmol substrate, 500 mg of SILP20 (5 mg of **1**, 95 mg of IL, 400 mg of SILP[NTf_2_]); entry 11: taken from Ref.[7a]

Increasing the S/C ratio from 200 to 1000 resulted in an increase of TON and TOF values to 1000 and 1333 h^–^^1^, respectively, with quantitative conversion within 45 min (entries 5 and 10). In our previous study,[7a] this reaction was carried out under the same conditions using an imidazolium‐based IL modified silica gel as support and ([bm_2_im][NTf_2_]) as IL. In that case, a three‐fold faster conversion was observed (entry 11).

**Figure 6:**
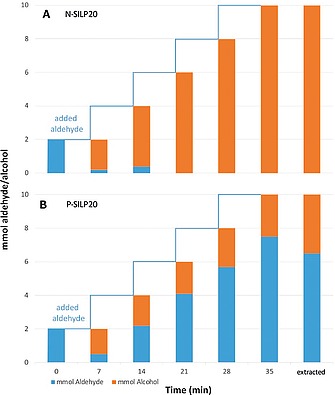
Catalyst recycling for the SILP hydrogenation of 4‐fluorobenzaldehyde in different ionic liquids. (A) [N_4441_][NTf_2_] and (B) [P_4441_][NTf_2_]. Conditions: 500 mg of N‐SILP20 (A) and P‐SILP20 (B), 2 mL *n*‐heptane, 2 mmol 4‐fluorobenzaldehyde/step, 0.5 mol‐% of catalyst **1**, 5 mol‐% of DBU, 25 °C, 50 bar H_2_.

In order to test the recyclability of the catalyst in this reaction, we reused the N‐SILP20 and P‐SILP20 systems by repeated addition of aldehyde after each reaction cycle (Figure 6). This procedure was carried out four times every 7 minutes. It was found that N‐SILP20 could be recycled yielding a TON of 1000 and a TOF of 1714 h^–^^1^, while P‐SILP20 lost almost completely its activity after the first cycle. This apparent poisoning of the catalyst dissolved in [P_4441_][NTf_2_] might be related to the presence of residual surface OH groups, which was indicated by a comparison of the ^29^Si NMR spectra of P‐SILP20 and N‐SILP20 (Figure 2). A similar effect was also observed in our previous study on the SILP‐catalyzed continuous formation of cyclic carbonates, were residual surface OH groups were responsible for the rapid loss of activity over time.^16^

N‐SILP20 and P‐SILP20 have also been tested with other substrates (Table 3) in the presence of 0.5 mol‐% of catalyst, 5 mol‐% of DBU at 25 °C under 50 bar hydrogen pressure and compared to the corresponding Im‐SILP20 system.[7a] It was found that under these reaction conditions, all three SILP20s could efficiently catalyze the hydrogenation of aromatic and heteroaromatic aldehydes bearing both electron‐withdrawing halogen groups such as F, Cl, and Br in 4‐halobenzaldehydes or electron‐donating substituent such as OMe in 4‐anisaldehyde.

**Table 3:**
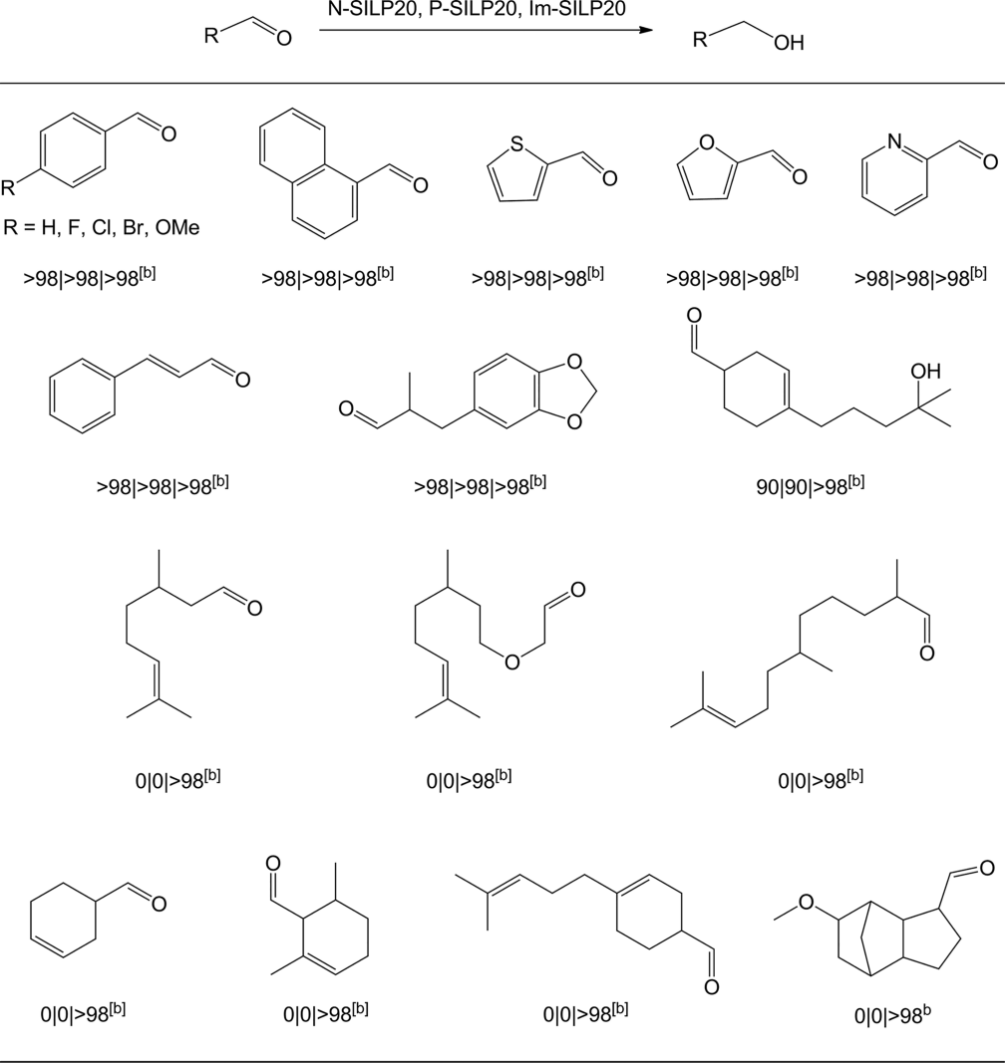
Achieved conversions in SILP hydrogenations of different aldehydes using catalyst 1 dissolved in different ionic liquids [N_4441_][NTf_2_] (N‐SILP), [P_4441_][NTf_2_](P‐SILP) and [bm_2_im][NTf_2_] (Im‐SILP)^a^

a Conditions: 500 mg of SILP20, 2 mL *n*‐heptane, 2 mmol substrate, 0.5 mol‐% **1**, 5 mol‐% DBU, 25 °C, 50 bar H_2_, 1 h.

Yields for N‐SILP20, P‐SILP20 and Im‐SILP20 determined by calibrated GC/MS and ^1^H NMR spectroscopy with mesitylene as internal standard.

High chemoselectivity was observed for the challenging α,β‐unsaturated cinnamaldehyde since its C=C double bond was not hydrogenated. Aldehydes with longer aliphatic chains or cycloalkyl substituents, however, which were quantitatively reduced using Im‐SILP20,[7a] showed no conversion with either the N‐SILP20 or the P‐SILP20 catalyst. This result is believed to be related to solubility differences and/or specific substrate/ionic liquid interactions. [bm_2_im][NTf_2_], for example, can interact through its aromatic [bm_2_im]^+^ cation with aromatic aldehydes via π–π bonding and with the nonaromatic aldehydes in Table 3, which all contain double bonds, which is currently further investigated in our group using experimental in‐situ spectroscopic methods in combination with semi‐empirical models (COSMO‐RS, Kamlet‐Taft).

## Conclusions

In the present study, the catalytic reduction of aldehydes to alcohols in the SILP mode – using an Fe^II^ PNP pincer complex dissolved in ionic liquid and applied as a thin film on silica gel – was investigated. Two different ionic liquids containing the same anion but different cations – [N_4441_]^+^ in N‐SILP and [P_4441_]^+^ in P‐SILP – were applied. The catalytic performance in terms of TON and TOF values for single reactions as well as for repeated reaction cycles with the same, recycled catalysts was determined and compared to a previous study of the same reactions using an imidazolium‐based ionic liquid (Im‐SILP). Aldehydes with aromatic or heteroaromatic substituents showed quantitative conversions with all three ionic liquids, although an about three times lower reaction rate for N‐SILP and P‐SILP compared to Im‐SILP. Aldehydes with larger alkyl or cycloalkyl groups, on the other hand, gave no conversion with N‐SILP and P‐SILP in contrast to quantitative conversion with Im‐SILP. Solubility effects and/or specific substrate/ionic liquid interactions are believed to be the main reason for these differences, although further studies are needed here. The N‐SILP and the P‐SILP systems gave very similar results for single reactions. Catalyst recycling, however, by repeated aldehyde addition worked well only with the N‐SILP catalyst, whereas the P‐SILP catalyst became inactive after the first reaction cycle, probably which caused by catalyst poisoning by residual surface hydroxyl groups of the silica support. The trends which were observed in the biphasic catalytic reactions, where [P_4441_][NTf_2_] as the solvent gave the highest reaction rates followed by [bm_2_im][NTf_2_] and [N_4441_][NTf_2_], were not replicated in the SILP mode, where 3‐times higher reaction rates were observed in [bm_2_im][NTf_2_] compared to [P_4441_][NTf_2_] and [N_4441_][NTf_2_]. As mentioned in the introduction, a complex interplay of different factors, which are still poorly understood until today, determines the performance of a SILP catalyst. Further studies and molecular information of the interactions between catalyst, substrates, and products at the reaction interface ionic liquid/organic solution are needed to understand and optimize this powerful catalytic technique.

## Experimental Section

**Materials:** All operations were performed under argon atmosphere by using Schlenk techniques or in an MBraun inert‐gas glovebox. The solvents were purified according to standard procedures.^17^ The deuterated solvents were purchased from Aldrich and dried with 4 Å molecular sieves. As support material for the SILP catalysts, silica gel (SG; silica gel 60, Carl Roth, 400–230 mesh) was used. The ionic liquids (tributylmethyl)ammonium bis(trifluoromethanesulfonyl)imide ([N_4441_][NTf_2_]) and tributylmethylphosphonium bis(trifluoromethanesulfonyl)imide ([P_4441_][NTf_2_]) were dried for at least 24 h at room temperature and 0.01 mbar before use and were stored under an argon atmosphere; resulting water contents were typically <500 ppm. The complex [Fe(PNP^Me^‐*i*Pr)(CO)(H)(Br)] (**1**) was prepared according to the literature.[5b] All aldehyde substrates were obtained from commercial sources and purified by distillation prior to use. Hydrogen (99.999 % purity) was purchased from Messer Austria and used as received.

**Synthesis of SILPs**

**N‐SILP[NTf_2_] and P‐SILP[NTf_2_]:** As a first step, silica gel was treated at 400 °C in an oven for 16 h, carefully evacuated and stored in the glovebox. 5 g of the treated silica gel, 2 g of 3‐iodopropyltrimethoxysilane, and 15 mL of toluene were refluxed for 48 h. The solvent was carefully removed under reduced pressure. The resulting SILP[I] was added to an extraction hull in the glovebox. It was brought to an evacuated 100 mL Soxhlet extractor and flushed with argon gas under a slight flow of argon. The SILP[I] was extracted with anhydrous ethanol for 16 h. The powder was dried under reduced pressure afterwards. Then, 2 g of tributylamine or tributylphosphine in 15 mL of toluene was added to 5 g of SILP[I] and refluxed for 48 h to prepare N‐SILP[I] and P‐SILP[I], respectively. The materials were extracted with anhydrous ethanol for 16 h. For ion exchange, the N‐SILP[I] and P‐SILP[I] were treated with 2 g of Li[NTf_2_] in 10 mL of CH_2_Cl_2_ at room temperature for 24 h. They were again extracted with a Soxhlet extractor as described before. N‐SILP[NTf_2_] and P‐SILP[NTf_2_] were dried under reduced pressure and stored in the glovebox.

**N‐SILP10–40 and P‐SILP10–40:** 5 mg of **1**, specific amount of N‐SILP[NTf_2_] or P‐SILP[NTf_2_] and 2 mL of anhydrous ethanol were added to a round‐bottomed flask equipped with a small stirring bar and a vacuum valve in the glovebox. The reaction mixture was stirred for 1 h. The solvent was carefully removed under reduced pressure. The powder was dried for an additional 5 min.

**Material Characterization**

The C, H, N, S content was double‐determined utilizing a Vario Macro elemental analyzer (CHNS‐mode, WLD). Solid‐state ^29^Si, ^13^C, and ^31^P CP‐MAS NMR measurements were recorded on a Bruker AVANCE 300 DPX spectrometer, equipped with a 5 mm broadband inverse probe head. Magic Angle Spinning was performed at 4 kHz spinning rate at 59.57 MHz for ^29^Si, at 75.40 MHz for ^13^C, and at 121.38 MHz for ^31^P NMR measurements. Textural properties were analyzed by N_2_ physisorption (ASAP 2020 Micrometics GmbH) using the Brunauer–Emmett–Teller (BET) theory for determining the surface area and the Barret–Joyner–Halenda (BJH) method for obtaining the pore size distribution. Infrared spectra were recorded with a Bruker Vertex 80 FTIR spectrophotometer using a narrow band MCT detector measuring diffuse reflectance. 256 Scans were collected for each spectrum with 4 cm^–1^ resolution. ^1^H, ^19^F{^1^H}, and ^31^P{^1^H} NMR spectra were recorded on Bruker AVANCE‐250 spectrometer. ^1^H spectra were referenced internally to residual protic‐solvent resonances and are reported relative to tetramethylsilane (*δ* = 0 ppm). ^31^P{^1^H} NMR spectra were referenced externally to H_3_PO_4_ (85 %) (*δ* = 0). ^19^F{^1^H} spectra were referenced externally to trifluorotoluene (0.05 %) (*δ* = 0 ppm). GC–MS analyses were recorded on an ISQ LT Single quadrupole MS (Thermo Fisher) directly connected to a TRACE 1300 Gas Chromatograph (Thermo Fisher), using a Rxi‐5Sil MS (30 m, 0.25 mm ID) cross‐bonded dimethyl polysiloxane capillary column. Fe leaching was monitored using an inductively coupled plasma (ICP) optical emission spectrometer PerkinElmer OPTIMA 8300 equipped with an SC‐2 DX FAST sample preparation system. A customized single‐element (Merck, Roth) standard was used for the calibration. All samples were extracted using ethanol and methanol (two times each), followed by solvent‐evaporation and acid‐digestion (HNO_3_ and H_2_O_2_ at a 2:1 ratio).

**General Procedure for the Hydrogenation of Aldehydes**

All hydrogenation reactions were carried out in a Carl Roth 100 mL stainless steel autoclave containing a 20 mL glass vial equipped with a stirring bar. In the glovebox, freshly prepared SILP20 was added to the glass vial which was sealed with a septum afterwards. 2 mmol of the substrate, 5 mol‐% DBU and 2 mL *n*‐heptane were mixed and taken up in a syringe. The vial was added to the autoclave, the septum was removed and the autoclave was evacuated and flushed 3 times with argon gas. The solution was injected into the autoclave under a slight flow of argon. The autoclave was then purged three times with hydrogen gas before the final pressure was adjusted to the specified value and the reaction was carried out for the stated time. Afterward, the hydrogen gas was released and the vial was taken out of the autoclave. The reaction mixture was three times extracted with 1.5 mL of diethyl ether, decanted and filtered through aluminum oxide. The solvent was then slowly removed under reduced pressure. The residue was analyzed by ^1^H and/or ^19^F{^1^H} NMR spectroscopy and yields were determined by integration of the aldehyde and alcohol signals and referenced to mesitylene. If the yields were detected by GC–MS, one drop of the extracted reaction solution was taken out and filled up with 1 mL of CH_2_Cl_2_ and injected via a 4 mm syringe filter (PTFE membrane, 0.2 µm pore size) into a GC–MS vial which was then sealed with a septum.

## References

[1] a) H.‐P. Steinrück and P. Wasserscheid , Catal. Lett., 2015, 145, 380–397;

[2] a) R. Hayes , G. W. Gregory and R. Atkin , Phys. Chem. Chem. Phys., 2010, 12, 1709–1723; 20145835 10.1039/b920393a

[3] a) M. T. Heinze , J. C. Zill , J. Matysik , W. D. Einicke , R. Gläser and A. Stark , Phys. Chem. Chem. Phys., 2014, 16, 24359–24372; 25300707 10.1039/c4cp02749c

[4] a) S. Shylesh , D. Hanna , S. Werner and A. T. Bell , ACS Catal., 2012, 2, 487–493;

[5] a) N. Gorgas and K. Kirchner , Acc. Chem. Res., 2018, 51, 1558–1569; 29863334 10.1021/acs.accounts.8b00149 PMC6011182

[6] S. Weber , J. Brünig , V. Zeindlhofer , C. Schröder , B. Stöger , A. Limbeck , K. Kirchner and K. Bica , ChemCatChem, 2018, 10, 4386–4394. 30450132 10.1002/cctc.201800841 PMC6221069

[7] a) J. Brünig , Z. Csendes , S. Weber , N. Gorgas , R. W. Bittner , A. Limbeck , K. Bica , H. Hoffmann and K. Kirchner , ACS Catal., 2018, 8, 1048–1051;

[8] S. R. Wasserman , G. M. Whitesides , I. M. Tidswell , B. M. Ocko , P. S. Pershan and J. D. Axem , J. Am. Chem. Soc., 1989, 111, 5852–5861.

[9] L. F. Bobadilla , T. Blasco and J. A. Odriozola , Phys. Chem. Chem. Phys., 2013, 15, 16927–16934. 24002208 10.1039/c3cp52924j

[10] J. A. Riddick , W. B. Bunger and T. K. Sakano , in: Organic solvents: physical properties and methods of purification,, 4th ed., Volume II, Wiley, New York, 1985, pp. 639.

[11] M. M. Rahman , H. Y. Liu , K. Eriks , A. Prock and W. P. Giering , Organometallics, 1989, 8, 1–7.

[12] J. P. Blitz , R. S. S. Murthy and D. E. Leyden , J. Colloid Interface Sci., 1988, 126, 387–392.

[13] a) K. Motokura , S. Itagaki , Y. Iwasawa , A. Miyaji and T. Baba , Green Chem., 2009, 11, 1876–1880;

[14] E. Finocchio , E. Macis , R. Raiteri and G. Busca , Langmuir, 2007, 23, 2505–2509. 17243745 10.1021/la062972b

[15] K. Hanke , M. Kaufmann , G. Schwaab , M. Havenith , C. T. Wolke , O. Gorlova , M. A. Johnson , B. Prasad Kar , W. Sander and E. Sanchez‐Garcia , Phys. Chem. Chem. Phys., 2015, 17, 8518–8529. 25749545 10.1039/c5cp00116a

[16] A. Sainz Martinez , C. Hauzenberger , A. R. Sahoo , Z. Csendes , H. Hoffmann and K. Bica , ACS Sustainable Chem. Eng., 2018, 6, 13131–13139.

[17] D. D. Perrin and W. L. F. Armarego , in: Purification of Laboratory Chemicals, 3rd ed.; Pergamon, 1988.

